# Giant pericardial lipoma arising from the right ventricular outflow tract

**DOI:** 10.1093/ehjcr/ytag437

**Published:** 2026-06-10

**Authors:** Min-Yuan Liu, Nai-Hsin Chi

**Affiliations:** Department of Surgery, National Taiwan University Hospital, College of Medicine, National Taiwan University, No. 7, Chung-Shan South Road, Taipei 100, Taiwan; Department of Surgery, National Taiwan University Hospital, College of Medicine, National Taiwan University, No. 7, Chung-Shan South Road, Taipei 100, Taiwan; Graduate Institute of Clinical Medicine, College of Medicine, National Taiwan University, Taipei, No. 7, Chung-Shan South Road, Taipei 100, Taiwan

**Keywords:** Pericardial lipoma, Cardiac tumour, Right ventricular outflow tract, Cardiac magnetic resonance imaging, Cardiac computed tomography, Surgical resection

## Case description

**Figure 1:**
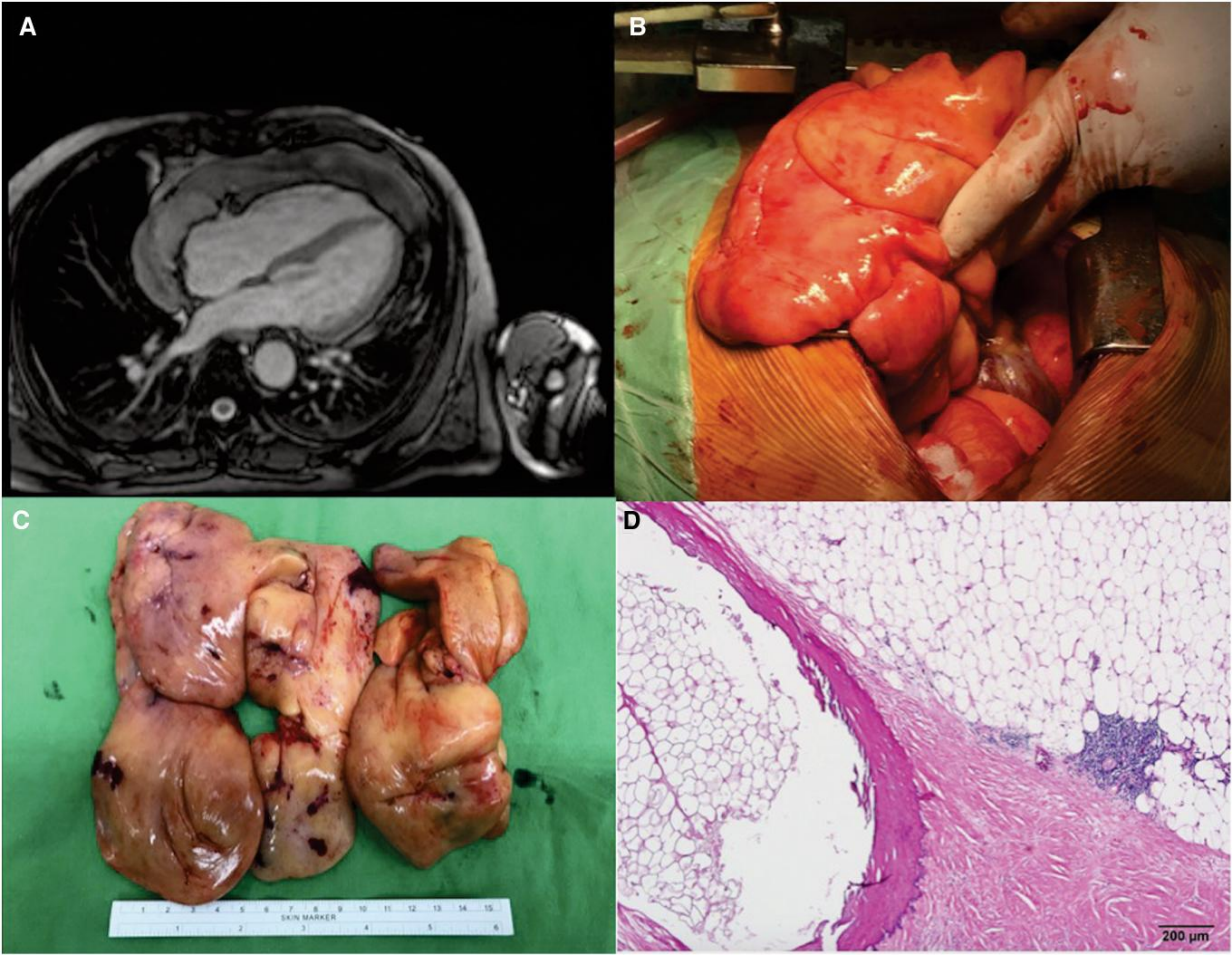
Multimodality imaging, intraoperative, and histopathological findings of a giant pericardial lipoma arising from the right ventricular outflow tract. (*A*) Cardiac magnetic resonance imaging (axial view) demonstrating a large homogeneous pericardial mass with signal characteristics consistent with adipose tissue. (*B*) Intraoperative photograph showing the well-demarcated lipomatous mass upon exposure via median sternotomy. (*C*) Gross specimen of the resected lipoma measuring 4 × 20 × 18 cm and weighing 2850 g. (*D*) Histopathological examination (haematoxylin and eosin staining; scale bar = 200 μm) revealing mature adipose tissue composed of uniform adipocytes with interspersed fibrous septa, without cytologic atypia or malignant features.

A 57-year-old man presented with a 2-year history of intermittent right-sided chest tightness, and recurrent syncope. Computed tomography demonstrated extensive pericardial adipose tissue. Cardiac magnetic resonance imaging revealed a giant pericardial mass with homogeneous signal characteristics consistent with adipose tissue (*Figure 1A*), suggestive of a benign lipomatous lesion.^1,2^

Elective surgical resection was performed via median sternotomy. Intraoperatively, a large, well-demarcated mass originating from the right ventricular outflow tract (RVOT) was identified and completely excised (*Figure 1B*). The resected specimen weighed 2850 g and measured 4 × 20 × 18 cm (*Figure 1C*). Histopathological examination demonstrated mature adipose tissue composed of uniform adipocytes without cytologic atypia or malignant features, confirming the diagnosis of cardiac lipoma (*Figure 1D*). The postoperative course was uncomplicated, and the patient remained asymptomatic at follow-up.

## Supplementary Material

ytag437_Supplementary_Data

## Data Availability

The data underlying this article are available in the article.

## Notes

**Consent:** Written informed consent for publication was obtained from the patient.
